# Minutes of the Central New York Homœopathic Medical Society, December, 1883

**Published:** 1884-03

**Authors:** C. P. Jennings

**Affiliations:** Secretary


### FROM THE MINUTES OF A MEETING OF THE CENTRAL NEW YORK HOMŒOPATHIC MEDICAL SOCIETY IN DECEMBER LAST.

Dr. C. L. Swift read a paper on "Anæsthetics in Labor." Accepted with thanks.

Dr. Hawley—I like the paper very much. It is wonderful that there should be need to read such a paper on the subject before a homœopathic society. Yet it is necessary. The perils of labor are greatly enhanced by anæsthetics, especially the hæmorrhage.

Dr. Bigelow—Have known homœopathists to use anæsthetics freely. I do not use them.

Dr. Harris—Have used them a very few times in severe pain, after careful examination, and with good success. We should be posted on the provings of anæsthetics. The people need the publication of such a paper as Dr. Swift's.

Dr. Hawley—Feel as Dr. Harris does about the publishing of the paper. This Society seeks to promote the intelligence and usefulness of its members. In all schools there is a tendency to discontinue the use of anæsthetics in labor. Nature is to be relied upon. The homœopathist has the means of meeting disturbances in labor according to law. Have had experience with anæsthetics in labor in three cases. In all of them the labor was protracted two or three days after using anæsthetics, and there followed headaches of unprecedented intensity. Had a case where a woman had taken an anæsthetic in former labors. She desired that I would use it. I refrained. She thanked me afterward for not giving it to her.

Dr. Martin—I want the woman to be thoroughly alive, so as the better to avoid mistake. The after effects of the anæsthetic on the milk and on the child are undesirable. Can always dissuade the patient from taking anæsthetics.

Dr. Harris—Am always averse to anæsthetics and to instruments, except in extreme cases. Have seen worse effects from instruments than from anæsthetics.

Dr. Nash—Have never used an anæsthetic in labor. Like whisky, it would be an expensive luxury, a hurtful luxury. I say this to my patients. I assure them of my willingness to use anæsthetics if necessary; but I do not find it necessary to resort to them. When the labor is over, invariably the patient is glad that an anæsthetic was not used.

The death of Dr. Augustus Poole, of Oswego, was noted, and a minute of sympathy with his bereaved family was adopted. Dr. Poole was one of the organic members of this Society, and always an honored member.

Dr. Nash read a paper on "Our Journals." Accepted, with thanks.

Dr. Hawley—There must be a change in our journals and in our schools, else we will become an opprobrium. It is not true that the old-school pathology is superior to ours. Have now in my care a case of scirrhus. Breast swollen and tender just before menstruation, and the tumor in the breast becomes then more troublesome. It is the right breast. Nipple retracted slightly. Gave Conium. No result. Learned that she had a fall some three years ago, and struck upon the breast on the side of a chair. Gave Arnica CM, one dose, three weeks ago. The lobulated condition of the tumor is gone. Improvement moves on apace. Clearly this is a case of scirrhus. Any allopathic authority would have pronounced it to be scirrhus, and he would have advised amputation. Amputation would, in two years, be followed by a funeral.

Dr. Seward—Cured a case of scirrhus thirty years ago. It remains cured to this day. We should not allow our diagnosis of scirrhus to be denied because the scirrhus has been cured.

Dr. Nash read a proving of Lac caninum. The paper was accepted with thanks. C. P. Jennings, *Secretary.*

